# Cancer-induced immunosuppression can enable effectiveness of immunotherapy through bistability generation: A mathematical and computational examination

**DOI:** 10.1016/j.jtbi.2020.110185

**Published:** 2020-05-07

**Authors:** Victor Garcia, Sebastian Bonhoeffer, Feng Fu

**Affiliations:** a Institute of Applied Simulation, Zurich University of Applied Sciences, Einsiedlerstrasse 31a, 8820 Wädenswil, Switzerland; b ETH Zurich, Universitätstrasse 16, 8092 Zürich, Switzerland; c Institute for Social and Preventive Medicine, University of Bern, Finkenhubelweg 11, 3012 Bern, Switzerland; d Department of Biology, Stanford University, 371 Serra Mall, Stanford CA 94305, USA; e Department of Mathematics, Dartmouth College, 27 N. Main Street, 6188 Kemeny Hall, Hanover, NH 03755-3551, USA

**Keywords:** Cancer, Mathematical modeling, Immunotherapy, Cancer-immune system interactions

## Abstract

Cancer immunotherapies rely on how interactions between cancer and immune system cells are constituted. The more essential to the emergence of the dynamical behavior of cancer growth these interactions are, the more effectively they may be used as mechanisms for interventions. Mathematical modeling can help unearth such connections, and help explain how they shape the dynamics of cancer growth. Here, we explored whether there exist simple, consistent properties of cancer-immune system interaction (CISI) models that might be harnessed to devise effective immunotherapy approaches. We did this for a family of three related models of increasing complexity. To this end, we developed a base model of CISI, which captures some essential features of the more complex models built on it. We find that the base model and its derivates can plausibly reproduce biological behavior that is consistent with the notion of an *immunological barrier*. This behavior is also in accord with situations in which the suppressive effects exerted by cancer cells on immune cells dominate their proliferative effects. Under these circumstances, the model family may display a pattern of *bistability*, where two distinct, stable states (a cancer-free, and a full-grown cancer state) are possible. Increasing the effectiveness of immune-caused cancer cell killing may remove the basis for bistability, and abruptly tip the dynamics of the system into a cancer-free state. Additionally, in combination with the administration of immune effector cells, modifications in cancer cell killing may be harnessed for immunotherapy without the need for resolving the bistability. We use these ideas to test immunotherapeutic interventions *in silico* in a stochastic version of the base model. This bistability-reliant approach to cancer interventions might offer advantages over those that comprise gradual declines in cancer cell numbers.

## Highlights

• The presence of an immunological barrier in cancer- immune system interaction (CISI) is consistent with the bistability patterns in that system.
• In CISI models, bistability patterns are consistent with immunosuppressive effects dominating immunoproliferative effects.
• Bistability could be harnessed to devise effective combination immunotherapy approaches.

## 1 Introduction

**Fig. 1:**
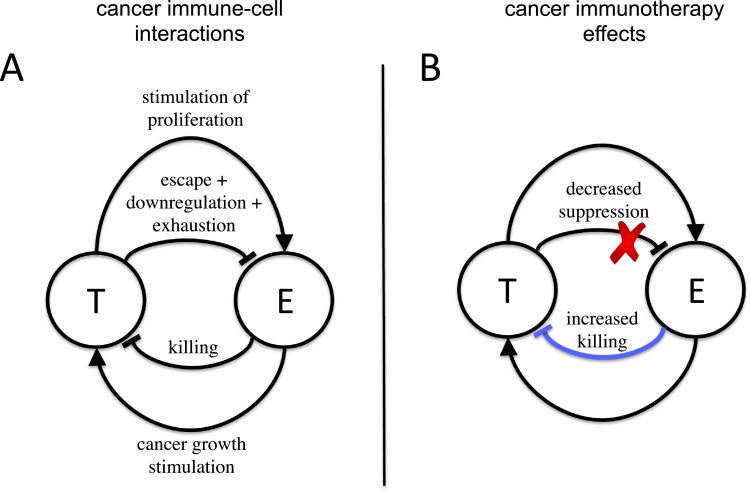
**Cancer-immune system interactions and effects of immunotherapy**. A) Interactions governing the dynamics between cancer cells (T) and immune cells targeting the cancer cells —termed effector cells— (E). A complex web of interactions has been identified (Mellman, Coukos, Dranoff, 2011, Eftimie, Bramson, Earn, 2010, Altrock, Liu, Michor, 2015), with both cell types capable to both stimulate and suppress each others’ growth. B) Immunotherapy acts by either increasing the killing rate of effector cells, for example by administrating new effector cells into the host (*adoptive T cell transfer*), or by impairing the escape mechanisms cancer cells adopt to avoid being cleared, for example by *monoclonal antibody therapy*.

Mathematical modeling of cancer-immune system interactions (CISI) can reveal the fundamental mechanisms that govern the dynamics of tumor growth (Altrock, Liu, Michor, 2015, Eftimie, Bramson, Earn, 2010), and represent and important tool to devise and test new forms of immunotherapy *in silico* (Talkington et al., 2018). The modeling relies on the appropriate integration of how cancer and immune cells affect one another (De Boer, Hogeweg, Dullens, De Weger, Den Otter, 1985, de Pillis, Radunskaya, Wiseman, 2005, Goldstein, Faeder, Hlavacek, 2004, Kronik, Kogan, Vainstein, Agur, 2008, Kuznetsov, Makalkin, Taylor, Perelson, 1994). Recent studies have uncovered a plethora of interactions by which cancer cells affect immune cells, and vice versa (Mellman, Coukos, Dranoff, 2011, Eftimie, Bramson, Earn, 2010). For instance, cancer cells elicit immune responses by a variety of effector cells (Parish, 2003, Smyth, Godfrey, Trapani, 2001, Mellman, Coukos, Dranoff, 2011). These effector cells, in particular white blood cells, natural killer cells (NKs) and cytotoxic T lymphocytes (CTLs) can lyse cancer cells (Quesnel, 2008), inhibiting tumor growth or even eliminating microscopic tumors altogether — a process termed *immunosurveillance* (Burnet, 1957, Burnet, 1967). However, cancers have also been shown to be able to suppress the proliferation of effector cells, which typically target cancer cells with specific biochemical signatures (Kooi, Zhang, Patenia, Edwards, Platsoucas, Freedman, 1996, Hamanishi, Mandai, Iwasaki, Okazaki, Tanaka, Yamaguchi, Higuchi, Yagi, Takakura, Minato, Honjo, Fujii, 2007). Cancer cells accrue mutations that, by changing these signatures, enable them to partially evade immune recognition (Altrock, Liu, Michor, 2015, Parsa, Waldron, Panner, Crane, Parney, Barry, Cachola, Murray, Tihan, Jensen, Mischel, Stokoe, Pieper, 2007, Hanahan, Weinberg, 2011). Furthermore, cancers may actively downregulate immune responses elicited against them (Keir, Butte, Freeman, Sharpe, 2008, Mellor, Munn, 2004, Aggarwal, Pittenger, 2005, Munn, Mellor, 2004, Marigo, Dolcetti, Serafini, Zanovello, Bronte, 2008), for example by recruiting the action of T regulatory cells (Mellman, Coukos, Dranoff, 2011, Ohta, Gorelik, Prasad, Ronchese, Lukashev, Wong, Huang, Caldwell, Liu, Smith, Chen, Jackson, Apasov, Abrams, Sitkovsky, 2006, Facciabene, Peng, Hagemann, Balint, Barchetti, Wang, Gimotty, Gilks, Lal, Zhang, Coukos, 2011), leading to *immunosupression*. A summary of these interactions shows that all combinations of stimulation and suppression on growth between cancer and immune cells may act simultaneously (see Fig. 1A). These interactions direct the interplay between the cancer and the immune system. Thus, their integration into mathematical models can reveal how immunotherapeutic approaches may be employed with maximum efficiency. The main immunotherapy approaches today work by impairing mechanisms that allow cancers to suppress immune action or by the administration of effector cells to the host (Dougan, Dranoff, 2009, Mellman, Coukos, Dranoff, 2011) (see Fig. 1B).

Due to their importance for immunotherapy, mathematical models of cancer-immune interactions have been the focus of intense theoretical efforts over the last decade (Altrock, Liu, Michor, 2015, Eftimie, Bramson, Earn, 2010). At the heart of this effort lies the task of identifying what features of cancer-immune dynamics are most effectively used to achieve immunotherapeutic success, i.e. cancer eradication. Following this tradition, we focus on deterministic, population based non-spatial models for analysis. We do this for two reasons. First, their mathematical simplicity is better suited to unambiguously reveal those model properties that are crucial for the modification of a cancer’s state, since the number of factors influencing the dynamics is manageable and understandable (Eftimie et al., 2010). Second, we can draw from a broad body of theory about immune system pressures developed within cancer research (Kuznetsov, Makalkin, Taylor, Perelson, 1994, Kuznetsov, Knott, 2001, d’Onofrio, 2005, d’Onofrio, 2008, Eftimie, Dushoff, Bridle, Bramson, Earn, 2011, Eftimie, Bramson, Earn, 2010, Lopez, Seoane, Sanjuan, 2014) as well as in virus dynamics, and in particular, human immunodeficiency virus (HIV) dynamics research (Nowak, May, 2000, Wodarz, 2007, De Boer, 2007, Althaus, De Boer, 2008, Conway, Perelson, 2014, Garcia, Regoes, 2015, Graw, Regoes, 2009, Graw, Richter, Oxenius, Regoes, 2011, Yates, Graw, Barber, Ahmed, Regoes, Antia, 2007, Althaus, De Boer, 2008, Elemans, Florins, Willems, Asquith, 2014, Garcia, Richter, Graw, Oxenius, Regoes, 2015, Regoes, Barber, Ahmed, Antia, 2007). To explore immunotherapeutic interventions *in silico*, we use a stochastic version of one of the models analyzed.

In this study, we have explored whether there exist consistent properties of cancer-interaction models that facilitate effective immunotherapy approaches. We investigated this question in a family of three related models of increasing complexity. To this end, we first developed a base model of cancer immune system interaction that captures some essential features of more complex models. The purpose of the model is to gain qualitative insights and then to serve as a guide for treatment therapies based on immune action. We analyzed under what parameter regimes the model produces biologically plausible behavior, and investigated how steady states are affected by changes in these parameters. We then successively extended the model to first include more complex features of cancer-immune system interactions (Conway and Perelson, 2014), such as saturating proliferative stimulation and exhaustion and in a second step, to include the action of NKs and CTLs. The common properties identified in these models were then used to study how combinations of immunotherapeutic treatments may work together to achieve eradication. To this end, we implemented stochastic simulations of the base model and analyzed how the dynamics are affected by *adoptive T cell transfer* (Rosenberg, 1991, Rosenberg, Yang, Restifo, 2004, Dudley, Wunderlich, Robbins, Yang, Hwu, Schwartzentruber, Topalian, Sherry, Restifo, Hubicki, Robinson, Raffeld, Duray, Seipp, Rogers-Freezer, Morton, Mavroukakis, White, Rosenberg, 2002, Rosenberg, Restifo, Yang, Morgan, Dudley, 2008), as well as by the disruption of immune evasion mechanisms of the cancer through for example monoclonal antibody therapy (Mellman et al., 2011; Brahmer, Drake, Wollner, Powderly, Picus, Sharfman, Stankevich, Pons, Salay, McMiller, Gilson, Wang, Selby, Taube, Anders, Chen, Korman, Pardoll, Lowy, Topalian, 2010).

The R code used to produce the figures of this manuscript, as well as to run the computations and stochastic simulations, is publicly available under https://doi.org/10.6084/m9.figshare.11536824.v1.

## 2 Materials and methods

To analyze the algebraic properties of a system of equations involving cancer-immune interactions, we used the program *Mathematica* (Wolfram Research, 2011). To find equilibrium points in situations where this was not algebraically possible, we used the *rootSolve* package in R (Soetaert, Herman, 2008, Soetaert, Soetaert, Petzoldt, Setzer, 2010).

Since all ordinary differential equations (ODEs) here described are deterministic, the time course of the decline of cancer cell numbers will always follow the same continuous trajectory given identical initial conditions. However, when small cancer cell numbers are reached, the temporal order at which the discrete events occur that underpin the dynamics will become important. Such events include the replenishment of immune cells and cancer cell deaths. Thus, at small cell numbers, accounting for the stochasticity of these events will add realism to the simulation, and help decide when eradication has effectively been achieved. To this end, we employed the Gillespie algorithm, where the interactions between cell types are explicitly simulated. Stochastic simulations of all ODEs were run in the R language for statistical computing (Team, 2012) by using the Gillespie algorithm (Gillespie, 1977) with tau leaping in the *adaptivetau* package (Johnson, 2011). If not stated otherwise, simulations were run with the set of parameter values given in Table 1. For alternative strategies to account for the stochasticity of CISI at the temporal mesoscale see (d’Onofrio, 2010).

**Table 1:** Standard parameter values for the base model.

Parameter	Description	Value
*a*	maximum replication rate of cancer cells (de Pillis et al., 2005)	0.514 ${\mathrm{day}}^{-1}$
*b*	inverse carrying capacity of tumor (de Pillis et al., 2005)	$1.02\cdot 10^{-9}$${\mathrm{cells}}^{-1}$
*k*	killing efficacy of immune cells (Ganusov, Goonetilleke, Liu, Ferrari, Shaw, McMichael, Borrow, Korber, Perelson, 2011, Wick, Yang, Corey, Self, 2005)	$10^{-4}-10$${\mathrm{cells}}^{-1}{\mathrm{day}}^{-1}$
*σ*	replenishment rate of immune cells (Althaus and De Boer, 2008)	10 $\mathrm{cells}\cdot {\mathrm{day}}^{-1}$
*d*	immune cell death rate (Ogg, Kostense, Klein, Jurriaans, Hamann, McMichael, Miedema, 1999, Casazza, Betts, Picker, Koup, 2001)	$1\cdot 10^{-2}$${\mathrm{day}}^{-1}$
*m*	maximum immune cell proliferation rate	$-10^{-6}$${\mathrm{day}}^{-1}$

To model treatment, two possible procedures were considered. First, an increase in the ability of immune cells to detect and eliminate cancer cells — their *killing rate or efficacy*. Second, adoptive immune cell transfer, corresponding to the injection of immune cells into the system (Rosenberg, 1991, Rosenberg, Yang, Restifo, 2004). Both of these mechanisms enhance the suppression of cancer growth by the immune system and can be applied in concert as *combination immunotherapy*. The time at which the killing rate is first enhanced, the treatment time *τ_k_*, can differ from the time at which cancer-specific immune cells are first injected into the system *τ_E_*. Also, the time period during which each of these treatment approaches are administered can vary, with Δ*τ_k_* the treatment period for killing efficacy enhancement, and Δ*τ_E_* the period for immune cell transfer.

We assume that treatment always consists of the administration of either immunoactivating compounds or immune cells into the host system, and we denote the amount of compound delivered as the administered *dose*. In the increased killing efficacy approach, we assume that the alteration induced by the administration of the compound is permanent, which is reflected in a change of the killing efficacy parameter of NK or CTLs, Δ*c*, or Δ*k*, respectively. The change occurs gradually over the time course of the treatment. For example, an initial CTL killing efficacy *k* before treatment initiation will by increased by Δ*k*/Δ*τ_k_* every day, leading to a final efficacy of k+Δk.

In the immune cell transfer approach the change in immune cell numbers is not permanent. Prior to the transfer, immune cells are assumed to be rendered ineffective at a predefined rate (for example by the shedding of NKG2D ligands such as MIC-A, MIC-B (Mellman et al., 2011) or alternatively, by the upregulation the ligands PD-L1 or PD-L2 (Kooi, Zhang, Patenia, Edwards, Platsoucas, Freedman, 1996, Hamanishi, Mandai, Iwasaki, Okazaki, Tanaka, Yamaguchi, Higuchi, Yagi, Takakura, Minato, Honjo, Fujii, 2007)). Once transferred into the system, immune cell numbers will be affected by already present cancer cells. Thus, immune cell numbers will change depending on the state of the cancer, because the cancer exerts a suppressive effect on immune cell proliferation. Conversely, cancer cell numbers will vary due to immune cell killing. Analogously to the dosage of killing efficacy increasing compounds, effector cells are administered at daily doses of Δ*E*/Δ*τ_E_* cells, until the full dose of Δ*E* has been dispensed.

## 3 Results

### Base-model of cancer-immune system interactions

We develop a base model of cancer-immune cell interaction. The model follows a large body of theory that uses two-equation deterministic ODEs to describe the interaction between cancer tumor cells and immune system cells (Kuznetsov, Makalkin, Taylor, Perelson, 1994, Kuznetsov, Knott, 2001, d’Onofrio, 2005, d’Onofrio, 2008, Eftimie, Dushoff, Bridle, Bramson, Earn, 2011, Eftimie, Bramson, Earn, 2010, Lopez, Seoane, Sanjuan, 2014). This model aims to replicate some basic features of cancer dynamics with a minimum of added complexity. With such an approach, qualitative insights about the behavior of the system can be obtained by relatively simple mathematical analysis. To this end, we make four fundamental assumptions. First, we assume the existence of immune cells, which are able to detect and kill tumor cells (Parish, 2003, Smyth, Godfrey, Trapani, 2001). These cells may eliminate microscopic tumors before they grow to endanger the organism; a process termed *immunosurveillance* (Burnet, 1957, Burnet, 1967). Second, these immune cells comprise the action of all cells that control tumor growth by antigen recognition and subsequent elimination (Eftimie et al., 2010), including natural killer (NK) cells (Khar, 1997) and CTLs (Boon and van der Bruggen, 1996) and are termed *effector cells*. The process by which the cancer cells are neutralized is called *lysis* (Quesnel, 2008). A background level of effector cells is present at all times (de Pillis and Radunskaya, 2003). Third, we assume that tumor growth is well described by a logistic growth in the absence of immune cells (Eftimie et al., 2010). Fourth, the interactions between tumor and cancer cells are governed by mass-action kinetics (Kuznetsov et al., 1994).

The third assumption of logistic growth warrants special discussion. The dynamics of tumor growth remain a debated issue in the literature. Benzekry et al. compared different theoretical growth dynamics in lung and breast tumor data of mice (Benzekry et al., 2014). They concluded that Gompertzian growth typically best predicts data. Gompertzian growth dynamics are motivated by the observation that tumor growth prior to detection appears to be faster than after detection (Eftimie et al., 2010). This suggests that the initial unbounded growth may be limited by the exhaustion of growth resources or cancer cell’s mutual growth impairment. Gompertzian growth dynamics, developed on the basis of birth and death processes, account for this behavior, and yield a sigmoidal type of growth curve for cancer cell numbers. However, the Gompertz model suffers from serious drawbacks, while other models did almost equally as well as the Gompertzian in predicting data (Benzekry et al., 2014). Because an upper bound of the proliferation rate is imposed by cell division time, the Gompertz model cannot adequately describe the dynamics of very small tumors (d’Onofrio, 2008, Eftimie, Bramson, Earn, 2010). Furthermore, theoretical analysis reveals that Gompertzian growth is at odds with the immuno-surveillance hypothesis, because the immune response is unable to eradicate cancers that grow in a Gompertzian fashion (Eftimie, Bramson, Earn, 2010, d’Onofrio, 2005). Thus, given these assessments, we chose a growth model that retains a sigmoidal cancer growth curve shape, namely logistic growth. With this, we preserve the notion of an exhaustion of growth resources. We note however, that alternative growth models may also successfully capture tumor growth patterns.

Our base model differs from most models in the literature in that it combines proliferative and suppressive effects of cancer cells on immune cells in one single term that describes its net effect. In this way, we can analyze how the systems behaves depending on the net effect of these opposing forces.

The equations for the base model are:

(1) $$\frac{dT}{dt}=aT\left(1-bT\right)-kTE,$$

(2) $$\frac{dE}{dt}=\sigma -dE+mET.$$

Tumor or cancer cells *T*, grow at a maximal rate *a* in a logistic fashion. The population density of the cancer cells is regulated by the coefficient *b*, which acts as an inverse carrying capacity. The cancer cells are detected and killed by effector cells *E* at a net rate *k* (Kuznetsov et al., 1994). We assume a constant supply of effector cells at a rate *σ* (Althaus, De Boer, 2008, De Boer, 2007), and a death rate *d* per effector cell (Wodarz, 2007). Effector cells can either be stimulated to proliferate or be impaired in their growth at a rate *m*, the net growth increase or decrease due to the presence of cancer cells. In other words, *m* can attain positive as well as negative values.

We proceed to analyze the possible equilibria of this system. We start by observing that $\left(T_{1}^{*},E_{1}^{*}\right)=\left(0,\sigma /d\right)$ is always a fixed point of the ODEs above. Two further solutions for T* can be represented by the quadratic formula (see Appendix A):

(3) $$T_{2,3}^{*}=\frac{a\left(m+bd\right)\pm \sqrt{\Delta_{s}}}{2abm},$$

where

(4) $$\Delta_{s}={\left(a\left(m+bd\right)\right)}^{2}-4abm\left(ad-k\sigma \right).$$

A closer inspection of the properties of the dynamics of (1), (2) reveals that all biologically relevant cases, namely those in which $T_{2,3}^{*}>0,$ are consistent with *m* < 0 (see Appendix A). This corresponds to a net immune cell proliferation suppression by cancer, which can arise by various mechanisms (Conway, Perelson, 2014, Keir, Butte, Freeman, Sharpe, 2008, Mellor, Munn, 2004, Aggarwal, Pittenger, 2005, Munn, Mellor, 2004, Marigo, Dolcetti, Serafini, Zanovello, Bronte, 2008, Kooi, Zhang, Patenia, Edwards, Platsoucas, Freedman, 1996, Hamanishi, Mandai, Iwasaki, Okazaki, Tanaka, Yamaguchi, Higuchi, Yagi, Takakura, Minato, Honjo, Fujii, 2007). *m* < 0 is also where a bistability pattern in the steady states of (1), (2) emerges. The alternative *m* > 0, leads to scenarios that are at odds with well established concepts of cancer modeling, and produce incomplete dynamics (see Appendix A). In particular, they conflict with the well-established notion of an *immunological barrier* (Kuznetsov et al., 1994); the idea that tumors have to grow above a critical threshold to reach a large size close to carrying capacity (Eftimie et al., 2010). Temporary changes in the activity of the immune system can lead to fluctuations in tumor size which place its size above the barrier, which then gives rise to cancer.

There are three possible cases of sign arrangements of the roots of (3) under *m* < 0: i) both negative, ii) one positive and one negative, iii) both positive. Scenario i) has $T_{1}^{*}$ as only biologically plausible solution. Only the cancer-free state exists. Case ii) signifies a single attractive equilibrium at a non-zero tumor size. The emergence of a cancerous cell suffices to ignite a replicative process that induces the establishment of a tumor close to carrying capacity 1/*b*. Thus iii), where $T_{2,3}^{*}>0,$ is the only case which admits stable equilibria compatible with an existing immunological barrier.

For $T_{2,3}^{*}>0$ to be satisfied, and while assuming that *a* > 0 and *b* > 0 for biological reasons, we obtain the following conditions (see Appendix A):

(5) m<0

(6) m+bd<0

(7) $$k>k_{l}\equiv \mathrm{ad}/\sigma$$

(8) $$k<k_{u}\equiv \left(\mathrm{ad}-\frac{{\left(a\left(m+\mathrm{bd}\right)\right)}^{2}}{4\mathrm{abm}}\right)\frac{1}{\sigma }$$

(9) $$=k_{l}-\left(\frac{{\left(a\left(m+bd\right)\right)}^{2}}{4abm}\right)\frac{1}{\sigma }.$$

**Fig. 2:**
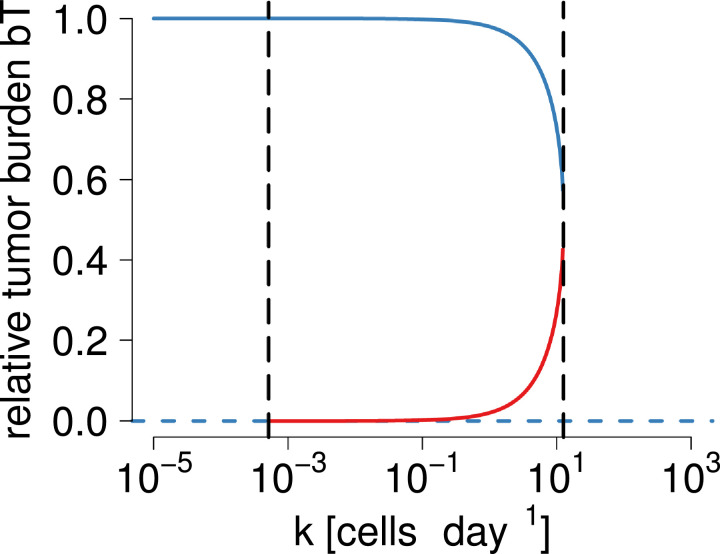
Bistability of immune control of tumor growth. Increasing values of *k* lead to the emergence of a bifurcation in equilibrium tumor cell numbers. The emergence of the bifurcations is indicated by black, vertical dashed lines. The upper blue line shows the largest stable steady states and the lower dashed blue line shows the smallest of the steady states, while red lines show unstable steady states for a given *k* value. If *bT* is one, the cancer has reached carrying capacity. At bT=0, no cancer cells exist. Thus, above a threshold value *k_u_*, the cancer is cleared. The parameter values are $m=-10^{-6},$σ=10 (Althaus, De Boer, 2008, De Boer, 2007), $d=2\times 10^{-2}$ (Ogg, Kostense, Klein, Jurriaans, Hamann, McMichael, Miedema, 1999, Casazza, Betts, Picker, Koup, 2001), a=0.514 (de Pillis et al., 2005), $b=1.02\times 10^{-9}$ (de Pillis et al., 2005). (For interpretation of the references to colour in this figure legend, the reader is referred to the web version of this article.)

These results reveal a bistability pattern that is mediated by the killing efficacy *k*. Fig. 2 shows how the increase in the parameter *k* leads to a bifurcation in the stable states of *T* and to bistability for *k*. At *k* < *k_l_* the system will reside in the aforementioned case ii). By increasing *k* above *k_l_* but below *k_u_*, the system will enter case iii), and move to i) as *k* > *k_u_*. In line with our expectations, values of *k* below the threshold value *k_l_* represent a similar situation as would be expected in the absence of immune cells: unchecked cancer growth. If *k* is gradually increased above *k_l_*, the tumor cells would have to begin replicating at increasingly large initial sizes in order to avoid being absorbed by the attractor at $T_{1}^{*}=0,$ that is, to be suppressed by the immune system. This is where the bifurcation appears, and now three equilibria, of which two are stable, dominate the dynamics. The parameter range spanned between *k_u_* and *k_l_*, $|k_{u}-k_{l}|=\frac{{\left(a\left(m+bd\right)\right)}^{2}}{4abm\sigma }$ is highly dependent on the ratio of *b* and *m*, as well as *σ*. Lastly, large values of *k* above *k_u_* entail that even few effector cells are able to clear the tumor, and even large tumors are eliminated with certainty.

At intermediate *k* (case iii) the base level of effector cells crucially affects the dynamics. In the absence of cancer, the equilibrium level of effector cells is at *σ*/*d*. The appearance of cancer cells will induce a killing process mediated by *k*. High enough effector cell levels will suppress tumor growth. In reality, the tumor might fluctuate above some threshold within which effector cells control it, and the suppression of further T cell proliferation (*m* < 0) by the cancer will dominate over the killing. Thus, the conditions (5), (6), (7), (8) mark the region of killing efficacy values that constitute an immunological barrier to the cancer. Increasing *k* values imply that this barrier is heightened: existing effector cells improve their capacity to completely eliminate the cancer.

This analysis may serve as a model to think about how to efficiently combine immunotherapy approaches. These results suggest that one mechanism to generate biologically plausible bistability is consistent with situations in which the cancer’s immunosuppressive effects outweigh its immunoproliferative effects (*m* < 0). The existence of a bifurcation implies that increasing killing efficacies will not simply gradually diminish cancer cell numbers. Instead, an increase above a critical value *k_u_* can terminally clear the cancer. Equally, if *k* is located in the range *k_l_* < *k* < *k_u_*, the adoptive transfer of effector cells may work by perturbing cancer cell numbers below the unstable equilibrium point $T_{2}^{*},$ after which it will be absorbed into $T_{1}^{*}=0$ and be cleared.

### Model variations

The here presented base-model, despite being strongly simplified, can help us intuitively understand more complex models of cancer-immune system interactions. In the following, we demonstrate that the basic bistability phenomenon can be replicated in two related, but more complex variations of the base-model. When possible, we also analyze whether multistability can arise, since it is relevant to *cancer dormancy*, and also because it might mitigate the effect of treatment.

### Incorporating saturation effect of tumor cells on immune response

A natural way to extend the base-model is to include more biologically plausible assumptions about the behavior of effector cells. Here, we retain the basic assumptions going into the base model, but refine the way that effector cells are reacting to the presence of tumor cells. In particular, we follow the approach by Conway et al., which allows for a very general set of behaviors of effector cells to arise and also provides estimates for the parameters (Conway and Perelson, 2014). This study is set in the context of HIV, where a great body of theory has been devoted to the particulars of CTL behavior (Wodarz, 2007). The equations are:

(10) $$\frac{dT}{dt}=aT\left(1-bT\right)-kTE,$$

(11) $$\frac{dE}{dt}=\sigma -dE+b_{e}\frac{T}{\kappa_{e}+T}E-d_{e}\frac{T}{\kappa_{d}+T}E.$$

Here, the interactions governing the rate of change in tumor cells have remained intact. Effector cell growth can now be stimulated at a maximum rate *b_e_* (Davenport, Ribeiro, Chao, Perelson, 2004, Conway, Perelson, 2014). The proliferation rate saturates with the number of tumor cells *T*, and is half-maximal at the Michaelis-Menten constant *κ_e_* (Conway and Perelson, 2014). In contrast, effector cells can be exhausted by contact with tumor cells, and die from its consequences at a maximal rate *d_e_* (Johnson, Kochin, McAfee, Stromnes, Regoes, Ahmed, Blattman, Antia, 2011, Conway, Perelson, 2014). As with proliferation, the effects of exhaustion – mediated by *κ_d_* – saturate. For typical values of these parameters see Table S1 in Appendix B. Models with a saturation term in $\frac{dT}{dt}$ have been analyzed before (Kirschner and Panetta, 1998), and have been thoroughly discussed in for example (Talkington et al., 2018).

As with the base model, $T_{1}^{*}=0$ is always a fixed point. The rest of the fixed points are determined by the roots of a cubic equation (see Appendix B). For the system of equations to generate bistability, that is, to give rise to exactly two positive equilibria in *T*, the following set of conditions needs to be satisfied (see Appendix B):

(12) $$18ABCD-4B^{3}D+B^{2}C^{2}-4AC^{3}-27A^{2}D^{2}>0$$

(13) $$\begin{array}{ccc} & & -2\sqrt{\frac{B^{2}-3AC}{9A^{2}}}\cos\left(\frac{1}{3}\arccos\left(\frac{B\left(2B^{2}-9AC\right)+27A^{2}D}{6A(B^{2}-3AC)}\sqrt{\frac{9A^{2}}{B^{2}-3AC}}\right)\right)\\ & & \;-\frac{B}{3A}<0 \end{array}$$

(14) $$\begin{array}{ccc} & & 2\sqrt{\frac{B^{2}-3AC}{9A^{2}}}\cos(\frac{1}{3}\arccos\left(\frac{B\left(2B^{2}-9AC\right)+27A^{2}D}{6A(3AC-B^{2})}\sqrt{\frac{9A^{2}}{B^{2}-3AC}}\right)\\ & & \;-\frac{2\pi }{3})-\frac{B}{3A}>0, \end{array}$$

where

(15) $$A=-ab\left(d-b_{e}+d_{e}\right),$$

(16) $$B=ab\left(b_{e}\kappa_{d}-d_{e}\kappa_{e}-d\left(\kappa_{d}+\kappa_{e}\right)\right)+a\left(d_{e}+d-b_{e}\right)-\sigma k,$$

(17) $$C=a\left(d_{e}\kappa_{e}-b_{e}\kappa_{d}\right)+ad\left(\kappa_{d}+\kappa_{e}-b\kappa_{d}\kappa_{e}\right)-\sigma k\left(\kappa_{d}+\kappa_{e}\right),$$

(18) $$D=\kappa_{d}\kappa_{e}\left(ad-\sigma k\right).$$

Analogously to the base model, a closer inspection of this result reveals that biologically plausible dynamics are consistent with $A=ab\left(b_{e}-d_{e}-d\right)<0$ (see Appendix B.1). Interestingly, this expression is independent of *κ_e_* or *κ_d_*. Since *a, b* > 0, this implies that $b_{e}<d+d_{e}$ which is analogous to the situation where *m* < 0 in the base model. The treatment rationale identified in the base model may therefore also be applicable for the extended base model with saturation (10), (11).

In Conway et al’s work (Conway and Perelson, 2014), this condition is satisfied by *b_e_* < *d_e_*, which is also functionally equivalent to *m* < 0 in our base-model: the effector cell population decreases due to cancer-mediated exhaustion. Importantly, the parameter choice in Conway et al. is also consistent with the emergence of bistable and multistable equilibira.

**Fig. A1:**
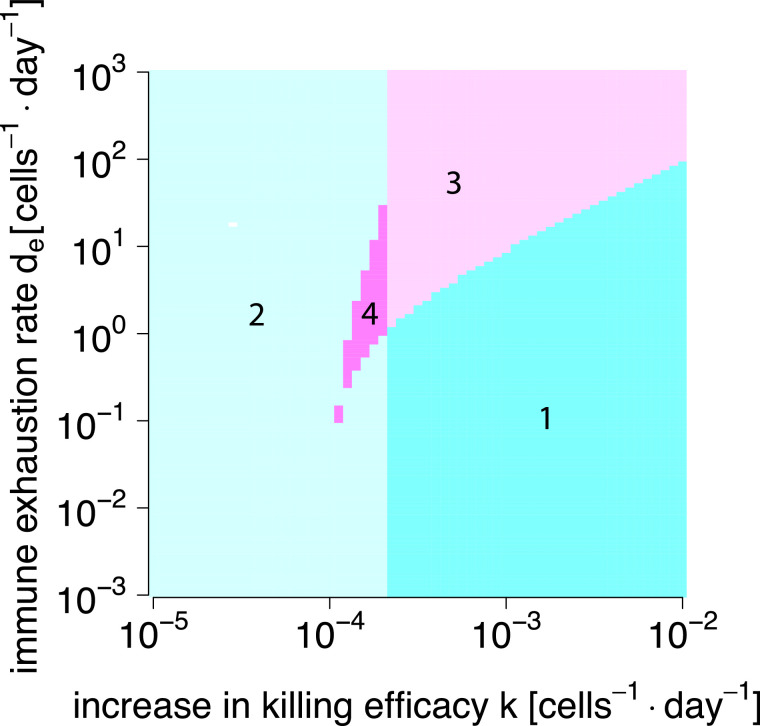
**Number of possible non-negative steady states of***T***of the model**(10), (11). Shown is the region of parameter space spanned by killing efficacy *k* and effector cell exhaustion *d_e_* divided into regions of different non-negative fixed points of the model (10), (11). The number of roots were identified numerically by means of the *rootSolve* package in R (Soetaert, Herman, 2008, Soetaert, Soetaert, Petzoldt, Setzer, 2010). Numbers indicate the number of non-negative fixed points in each parameter region. The region that supports multistability is colored in violet. The region that admits bistability (3 fixed points) is adjacent to the multistability region. The other used paramaters are as in Table 2.

Besides bistable patterns, the system (10), (11) can also generate a pattern of multistability. The conditions to obtain four equilibria in *T* for (10), (11) reads:

(19) $$18ABCD-4B^{3}D+B^{2}C^{2}-4AC^{3}-27A^{2}D^{2}>0$$

(20) $$\begin{array}{ccc} & & -2\sqrt{\frac{B^{2}-3AC}{9A^{2}}}\cos(\frac{1}{3}\arccos(\frac{B\left(2B^{2}-9AC\right)+27A^{2}D}{6A(B^{2}-3AC)}\\ & & \;\sqrt{\frac{9A^{2}}{B^{2}-3AC}}))-\frac{B}{3A}>0, \end{array}$$

where *A, B, C* and *D* are as in (15). Again, a biologically plausible arrangement of equilibrium points is in agreement with *A* < 0. Figure A1 shows that bistability becomes common for *d_e_* > 1 and $k>5\cdot 10^{-4},$ with the elimination of cancer ensuing after a critical *k*threshold is surpassed.

The existence of multiple stable equilibria may be interpreted as *cancer dormancy* (see *Discussion*). Cancer dormancy is the phenomenon of a period of non-growth of tumors. Often, this occurs in small, nearly undetectable tumors residing within body tissues (Wilkie, 2013). Fig. A1 shows that multistability is possible in (10), (11). The existence of multistability in two-equation models has been predicted and shown in other work (d’Onofrio, 2008, de Vladar, González, 2004, Kuznetsov, Makalkin, Taylor, Perelson, 1994). Here, we give precise analytical conditions for its emergence under (10), (11). When in a multistable regime, an increase in killing efficacy *k* might not directly lead to cancer eradication if treatment is started when the cancer is near carrying capacity 1/*b*. Instead, a new microscopic steady state (MISS, (d’Onofrio, 2008)) might be attained before a further increase in *k* leads to cancer clearance.

### Incorporating natural killer (NK) cells and tumor-specific CTL response

In a next step, we incorporated a further level of complexity by distinguishing between two types of effector cells: natural killer or NK cells, and cytotoxic T lymphocytes or CTLs. Models that account of the different roles between NK and CTL can be highly complex (de Pillis et al., 2005). To better understand where possible bistabilities originate from, we restrict ourselves to an extension of the base model with saturation, following an approach inspired by de Pillis et al. (2005) and Conway and Perelson (2014). Effector cells are now split into NK cells *N* and CTLs *E*:

(21) $$\frac{dT}{dt}=aT\left(1-bT\right)-cNT-kTE,$$

(22) $$\frac{dN}{dt}=\sigma -\mu N+b_{n}\frac{T}{\kappa_{bn}+T}N-d_{n}\frac{T}{\kappa_{dn}+T}N,$$

(23) $$\frac{dE}{dt}=-dE+b_{e}\frac{T}{\kappa_{be}+T}E-d_{e}\frac{T}{\kappa_{de}+T}E+\omega NT.$$

Here, cancer cells *T* are killed at rates *c* by NK cells, and at rates *k* by CTLs. The dynamics of the NK is now analogous to the dynamics of immune cells in (10), (11). We assume a constant supply of NK cells *σ* stemming from the host’s hematopoesis. NK cells die naturally at a rate *μ*. The maximum NK proliferation rate induced by the presence of cancer cells is *b_n_*, and the saturation coefficient is *κ_bn_*. Again, exhaustion occurs at a maximum rate *d_n_* and the saturation in *T* is half-maximal at *κ_dn_*. For CTLs, following (de Pillis et al., 2005), we assume that there is no constant supply of cells. Instead, CTL growth is stimulated by the NK-cancer cell interactions at a rate *ω*. This modeling approach ensures that CTLs are activated only after the emergence of the NK immune response (de Pillis et al., 2005). Several biological mechanisms appear to exist by which NKs can stimulate CTL growth (Pallmer and Oxenius, 2016). The work of Fan et al. suggests that already activated NK cells can facilitate the priming of CTLs by means of IFN-*γ* (Fan et al., 2006). The proliferation and exhaustion terms are as in the extended base model.

The model (21), (22), (23) can generate substantially more complex behavior than the two previously analyzed. As in the previous models (1), (2) and (10), (11), $T_{1}^{*}=0$ is always a fixed point. However, the NK-CTL model may allow for up to five other fixed points (see Appendix C). This is because finding the steady states of (21), (22), (23) can be reduced to finding the roots of the rate of change of *T*, $\frac{dT}{dt}\left(T\right),$ which is a polynomial of sixth order. The five fixed points besides $T_{1}^{*}=0,$ are the roots of a fifth order polynomial, for which no general solutions exist. Thus, we cannot draw similar conclusions for the existence of real fixed points as in the previous, relying on analogous conditions on discriminants Δ_*s*_ > 0 or Δ > 0. However, similarly to the base model (1), (2), a similar condition to *m* < 0 is compatible with biologically plausible arrangements of fixed point’s stabilities. The expression analogous to *m* < 0 is (see Appendix C):

(24) $$-ab\left(\left(b_{e}-d_{e}-d\right)\left(b_{n}-d_{n}-\mu \right)\right)-k\sigma \omega <0$$

The analogy to *m* < 0 arises from *k, σ, ω* > 0, valid bounds in most biological contexts. If tumor cells are able to exhaust NKs and CTLs, that is, if both $\left(b_{e}-d_{e}-d\right)<0$ and $\left(b_{n}-d_{n}-\mu \right)<0,$ then the system can display biologically reasonable behavior. Note that unlike the previous models, this condition can be satisfied by other means as well, such as increasing *k*. Again, the condition is independent of the four saturation coefficients *κ_be_, κ_de_, κ_bn_, κ_dn_*.

**Fig. A2:**
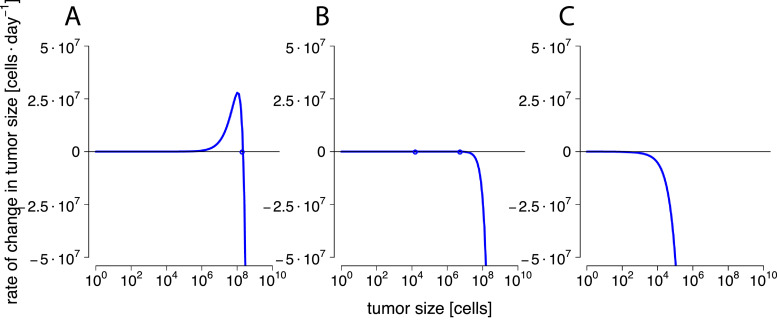
**Bistability of cancer cell fixed points induced by immune control of tumor growth in model**(21), (22), (23). Panels A) to C) show the function *dT*/*dt*(*T*) (blue lines) for different parameter values of *c. c* is varied from $10^{-7}$ (A) to $10^{-3}$ (B) 10^0^ (C). The blue points indicate the numerically found roots of *dT*/*dt*(*T*). Bistability emerges at $c=10^{-3}$. The parameter values were as specified in Table 3.

Due to the analytical unfeasability of the model (21), (22), (23), we resorted to numerical methods to prove the existence of basic bistability patterns (see Appendix C) (Soetaert, Herman, 2008, Soetaert). We found that the system is able to display bistability similar to that found in the extended base model with saturation (see Fig. A2).

### Bistability-based Strategies of Cancer Immunotherapy

The existence of bistability patterns in simple non-spatial cancer models as well as its variations, can be informative to the assessment of immunotherapeutic options and of their efficacy. Taking the base model (1), (2) as a foundation, three intervention approaches seem apparent. First, the elimination of the exhaustive effects of cancer on the immune cells (*m* < 0 → *m* > 0). Second, the increase of the killing efficacy of effector cells above some threshold (*k* < *k_u_* → *k* > *k_u_*). Third, the administration of effector cells (E→E+ΔE). In terms of the dynamics, this represents pushing the state of the system into the attraction basin of T=0. Combinations of these therapy approaches have previously been explored in simulations (Kirschner and Panetta, 1998).

In current immunotherapy, the two main available tools for cancer cell reduction correspond to the second (*antibody therapy*) and third (*adoptive T cell transfer*) options (Dougan, Dranoff, 2009, Mellman, Coukos, Dranoff, 2011). In antibody therapy, an increase in killing efficacy is attained by disrupting cancer cells' mechanisms for impairing T cell action. This impairment occurs by the acquisition of mutations in cancer cells that, for example, lead to the expression of the PD-L1 and PD-L2 ligands (Kooi, Zhang, Patenia, Edwards, Platsoucas, Freedman, 1996, Hamanishi, Mandai, Iwasaki, Okazaki, Tanaka, Yamaguchi, Higuchi, Yagi, Takakura, Minato, Honjo, Fujii, 2007). These ligands are known to bind to the PD-1 receptors on T cell surfaces, thereby downregulating the activation of the T cells. Thus, a direct effect of PD-1 immune checkpoint blockade is to halt cancer-induced T cell anergy and exhaustion (Mellman and Steinman, 2001). In this work we assume that ultimately, monoclonal antibodies binding to the ligands effectively increase *k* by interrupting this cancer escape mechanism. Possible increases in T cell recruiting and proliferation are neglected. In *adoptive T cell transfer* (Rosenberg, 1991, Rosenberg, Yang, Restifo, 2004), T cells are pre-programmed to kill host cells that carry particular biochemical signatures, for example certain peptides on their surface. The signatures are chosen such that they match characteristic features of cancer cells. Subsequently, these specific T cells are grown and injected into the blood stream of the patient (Mellman et al., 2011).

These two novel treatment methods can be combined to take advantage of the bistability phenomenon in cancer. We used the base model (1), (2) to investigate how a combination of both approaches could be used to clear the tumor, while accounting for the stochastic effects arising from singular cell-to-cell interactions. Starting from an already established tumor, increasing the efficacy of killing of effector cells will not by itself necessarily lead to the elimination of the tumor, unless very high levels of killing efficacy can be attained. Instead, increasing the killing efficacy by two orders of magnitudes will lead the system to equilibrate at tumor cell numbers lower than the carrying capacity (see Fig. 2). If the treatment with monoclonal antibodies has been sufficiently effective, it will have shifted the system into a regime with two stable equilibria, out of which one is the cancer-free state. If now the system is perturbed further with adoptive T cell transfer into the attraction basin of the cancer-free equilibrium, the waning of the effects of the first treatment will not lead to the reemergence of the cancer. Thus, the generation of a temporary bistability in the system can be exploited to perturb it into a cancer-free state.

**Fig. 3:**
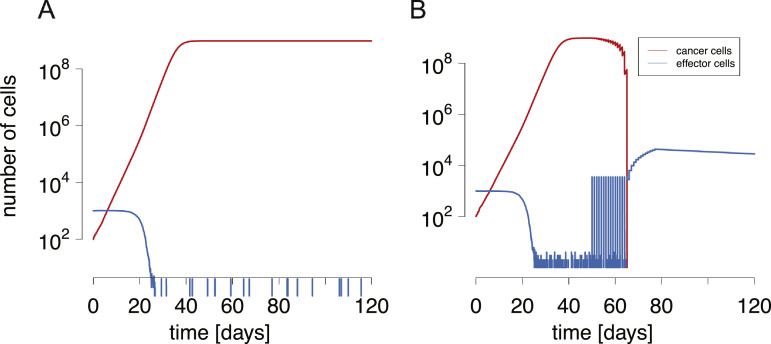
**Simulation runs of the base model without A) and with B) immunotherapeutic treatment.** A) Cancer replication begins at $T\left(0\right)=10^{2},$ and the natural equilibrium of the effectors is at $E\left(0\right)=10^{3}$. The cancer grows to carrying capacity in a time frame of around 45 days. B) Combined immunotherapeutic treatment is initiated at 50 days after the cancer has begun to grow. It lasts for 21 days in antibody therapy, and 28 days in adoptive cell transfer. Killing efficacies are increased to $\Delta k=10^{-1},$ while a total of $\Delta E=10^{5}$ cells are injected in a gradual fashion. Immune cells are eliminated rapidly by the powerful immune exhaustion effects exerted by the cancer cells. Parameter values are as in Table 1. In particular, $k=10^{-4}$.

To model the combined effects of killing efficacy increases by PD-1 specific monoclonal antibody and adoptive T cell transfer treatments, we used stochastic version of the base cancer immune interaction model (1), (2) (see *Materials and Methods*). Fig. 3 shows the time courses of the dynamics with and without treatment. Without treatment, a cancer that has surpassed the immunological barrier will grow unrestricted up to levels very close to carrying capacity (Fig. 3A). The combined administration of effector cells and killing efficacy-increasing compounds at first only gradually reduces cancer numbers (Fig. 3B). Daily administered effector cells Δ*E*/Δ*τ_E_* (*Materials and Methods*) can only temporarily affect the dynamics before they are rapidly suppressed and exhausted by the cancer (*m* < 0). When the state of the system is pushed into the attraction basin of the cancer-free state, cancer numbers rapidly go to zero. Further injections of effector cells become unnecessary, and effectors build up.

**Fig. 4:**
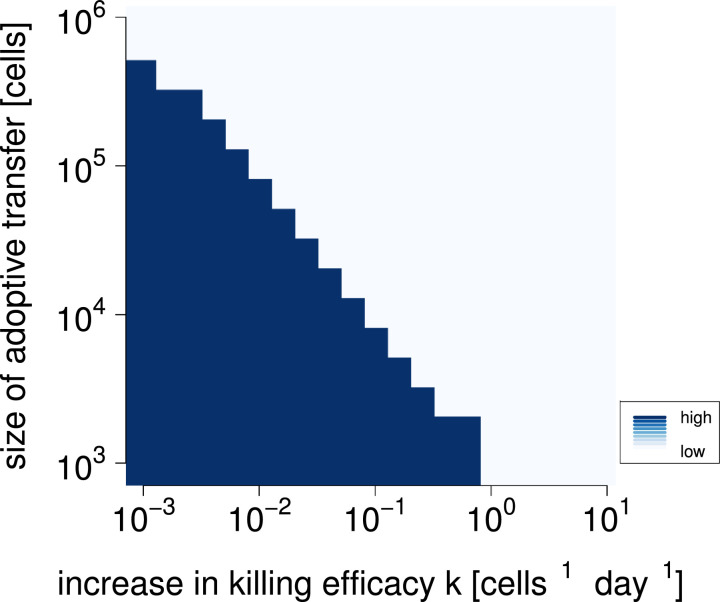
**Cancer cell numbers after combination immunotherapy after 120 days.** Immunotherapy begins at day 50, after the cancer has grown to full size, and lasts for a single day. Combination immunotherapy is implemented by increasing the killing efficacy of the effector cells and adoptive immune transfer of effector cells. Darker colors indicate high cancer cell numbers, implying that the cancer persists, while lighter colors indicate low cancer cell numbers. Parameter values are as in Table 1.

We then investigated whether an increase in *k* and the administration of effector cells *E* work together in a synergistic or antagonistic fashion to remove the tumor. Fig. 4 shows the outcome of combination immunotherapy, initiated simultaneously for antibody and T cell injections. Each immunotherapeutic approach may clear the cancer on its own. A marked frontier between cancer presence and clearance emerges. A lowering of killing efficacies along this frontier will lead to insufficient pressure to clear the cancer, but can be compensated by an increase in adoptive transfer doses. The linear shape of the frontier indicates that the two approaches do not mutually impair each others’ function.

## 4 Discussion

In this study, we have shown that the base model can only reproduce biologically plausible behavior if the suppressive effects exerted by cancer cells on immune cells dominate their proliferative effects. Under these circumstances, the base model displays a conspicuous pattern of *bistability*: The cancer-immune interaction dynamics gives rise to two distinct, stable states (a cancer-free, and a full-grown tumor state). Under bistability, the modification of the killing efficacy can lead to a bifurcation in cancer cell numbers, where the system may abruptly be tipped into a new, cancer-free state. Furthermore, in situations where exhaustion prevails over proliferation in immune cells, all analyzed models can produce bistability patterns that are biologically plausible. If this condition is not satisfied, the base model cannot produce biologically plausible behavior across a wide range of *k* values. We also formulated more complex extensions of the base model, which can generate *multistability*, a dynamic behavior that can be interpreted as stable microscopic cancers (d’Onofrio, 2005), or *cancer dormancy* (Kuznetsov, Makalkin, Taylor, Perelson, 1994, Wilkie, 2013). We gave the exact conditions under which multistability might arise in one model extension inspired by Conway and Perelson (2014). We also examined how bistability may be used for effective combination immunotherapy. We tested a combination of two different immunotherapeutic approaches in stochastic simulations of the base model. We found that the combination of treatment interventions is able to clear the cancer, and that the different treatment approaches do not impair one another.

How both treatment approaches investigated here would work in isolation has been studied in other work (d’Onofrio, 2008, Kuznetsov, Knott, 2001, Eftimie, Bramson, Earn, 2010). However, it is less apparent that they may be employed to work in concert without mutual impairment. This is important when considering side effects of these therapies. For example, the administration of PD-1 antibodies in mice have resulted in lung inflammation and cardiomyopathy (Nishimura, Okazaki, Tanaka, Nakatani, Hara, Matsumori, Sasayama, Mizoguchi, Hiai, Minato, Honjo, 2001, Nishimura, Nose, Hiai, Minato, Honjo, 1999, Mellman, Coukos, Dranoff, 2011). Thus, combination immunotherapy may help minimize the risks associated with standalone approaches.

Another advantage of this study lies in that it specifically shows how multistability may arise from standard assumptions about cancer-immune system interactions (saturation terms), and in that it gives precise conditions for its emergence. Although the first extended model with saturation (10), (11) is not intended to admit multiple steady states, these arise as a consequence of the basic assumptions about interactions.

Intermediate-sized cancers in multi-stable regimes may be interpreted as cancer dormancy (d’Onofrio, 2008, Kuznetsov, Makalkin, Taylor, Perelson, 1994, de Vladar, González, 2004), but the model (10), (11) does not explicitly explain how they may escape immune control. For immune escape to arise, additional processes must be assumed, such as stochastic perturbations or immunoediting (Wilkie and Hahnfeldt, 2013a). A discussion of how tumors might rise to large numbers in models very similar to the base model with saturation has been given in Kuznetsov et al. (1994) and by Wilkie and Hahnfeldt (2013b). In the Kuznetsov et al. model, a separatrix between the two main attraction basins passes close by the trivial, cancer-free steady state. When a cancer arises and starts to replicate at low numbers, it should follow a trajectory into a dormant, stable steady state. However, stochastic fluctuations can push the system’s state into the other attraction basin. In the Wilkie and Hahnfeldt model, the saddle point is the dormant state itself. Another of the main mechanisms hypothesized to drive the transition from dormancy to large tumors is *immunoediting* (Wilkie, 2013): The prolonged growth suppression of the tumor by the immune response leads to the selection of cancer mutations that escape immune pressure, effectively reducing the immune killing efficacy *k* Wilkie and Hahnfeldt (2013a). The model (10), (11) can also offer an intuitive explanation for this process, whereby a smaller, undetectable and stable equilibrium of cancer cells is maintained by a relatively weak immune response. The further decrease of the immune response efficacy by means of immune escape processes leads to the establishment of a full grown tumor. This is exemplified by the fact that decreasing *k* in Fig. A1 pushes the system into a region of parameter space where there exists a stable steady state for the tumor at carrying capacity, that is the full grown cancer state.

Most other models of cancer-immune interaction so far have attributed the phenomenon of dormant states to the existence of an additional compartment: quiescent cancer cells (Page, Uhr, 2005, Wilkie, 2013). These cells are assumed to replicate at a slower rate than normal cancer cells, and can revert back to a fast growing state by means of phenotypic switching or by acquiring further mutations (Wilkie, 2013). In line with other work, the model (10), (11) explains the existence of dormancy by a specific balance of cancer cell growth and killing attained in cancer-immune interactions, without relying on any additional compartments or assumptions. A notable example of how dormancy can emerge from cancer-immune interactions alone is given in Kuznetsov et al. (1994). A third mechanism for the emergence of dormancy has been given by Wilkie and Hahnfeldt (2013b). In this mechanism, dormant states are represented by saddle nodes traversed by a separatrix demarcating the adjacent attractor regions of either growth progression or tumor clearance.

This point emphasizes a last advantage of non-spatial ODE models: Understanding cancer growth requires an appropriate description of cancer-immune system interactions at multiple scales (Altrock et al., 2015). These scales range from cancer microenvironments to large numbers of already systemic cancers. ODE models offer useful tools to combine the behavior of both into a single framework (Eftimie et al., 2010), accounting for the frequency-dependent growth at early stages as well as the dominant immunosuppressive effects achieved by cancer when approaching carrying capacity levels.

A major caveat of the base model is that it cannot elicit an immune response. The elicitation of an immune response by a cancer, with a subsequent rise in effector cells, is an important aspect of cancer-immune system interactions. To achieve this, the base model would have to include a proliferation term for effectors that takes on different values than the suppression in the *T, E*-plane. The base model is thus more useful to study the aspects of how immunotherapy can be deployed to return the CISI system below an immunological barrier by external perturbation.

While simple modeling frameworks offer greater possibilities for an in-depth understanding, this approach also has its limitations. For instance, we have largely neglected stochastic attributes of cancer-immune system interactions in our mathematical analysis. These may mostly arise from the discreteness of cell-to-cell interactions, and are well captured by simulating the models by a Gillespie algorithm (Gillespie, 1977). We have addressed this shortcoming by adopting a stochastic simulation framework to implement immunotherapy. Other forms of stochasticity —for example the random accrual of malignant mutations in cancer cells— are also not explictly modeled. Instead, they are assumed to be captured by model parameter values. Environmentally based fluctuations (Eftimie, Bramson, Earn, 2010, Bose, Trimper, 2009), or changes in the exerted immune pressure due to, for example, disease (Mina et al., 2015), are also neglected.

The models here analyzed do also not account for spatial structure (discussed in more detail in Araujo, McElwain, 2006, Roose, Chapman, Maini, 2007, Chaplain, 2008). Spatial structure may change the way that effector cell killing affects cancer growth, as well as how the presence of cancer cells may mediate immune cell proliferation. In particular, we did not explore the fractional cell kill laws as introduced by de Pillis et al. (2005), hypothesized to account for some of the geometrical features of tumors (Lopez et al., 2014). In this approach, the total killing exerted by effectors *K*(*E, T*) is governed by the de Pillis-Radunskaya-Wiseman (PRW) law Lopez et al. (2014), where K(E,T)=D(E,T)T and $D\left(E,T\right)=d\frac{E^{\lambda }}{sT^{\lambda }+E^{\lambda }}$. Structurally, how (de Pillis et al., 2005) implement the recruitment of NK cells differs only slightly from our implementation in (21). However, how CTLs are recruited differs in structure from the simpler terms analyzed here in our models. With *λ* < 1 (obtained from model fits to mouse data (Lopez et al., 2014)), the behavior of the recruiting is qualitatively similar to that studied here: the recruiting of CTLs would then saturate with increasing cancer cell numbers *T*, but continue growing with increasing effector cell numbers *E*.

We thus assume that while the PRW law introduces an advantageous new concept in the modeling in tumor-immune system interactions, our deviating from it will not yield marked qualitative differences. Instead of attempting to capture tumor geometry behavior, the models analyzed here are rooted in the tradition of virus dynamics —especially HIV— which assumes well-mixed cell types (Nowak, May, 2000, Conway, Perelson, 2014). Thus, the PRW law seems to mainly address problems arising from tumor geometry, while this study focuses mainly on systemic cancer types —cancer types that do not manifest in single tumors only (Eftimie et al., 2010).

We have also not included the action of cytokines in our analysis, which are typically accounted for with a separate, additional equation (Arciero, Jackson, Kirschner, 2004, Kirschner, Panetta, 1998, Eftimie, Bramson, Earn, 2010). We have therefore not been able to assess the effectiveness of cytokine-based immunotherapy approaches in combination with the ones studied here. Models with cytokines display features like the persistence of large tumors, tumor dormancy, and tumor clearance upon immunotherapeutic treatment, as well as oscillations between these states (Eftimie, Bramson, Earn, 2010, Kirschner, Panetta, 1998). Including cytokines into a more comprehensive modeling framework would be an interesting topic for future work.

Our study has to be interpreted in the context of other models of cancer-immune system interactions. The most comprehensive mathematical analysis of two-equation models has been put forth by (d’Onofrio, 2005, d’Onofrio, 2008). d’Onofrio analyzed a generalized mathematical model in two variables —*x* denotes cancer and *y* effector cell densities—, deriving some general results on the existence of steady states and cancer eradication given some broad mathematical conditions on the interactions between these two cell types. Solutions were provided for the generalized model, but except for the rate of adoptive transfers *θ*(*t*) (where *t* is time), no specific dependence was given on how steady states change with parameter value modifications. Our base model corresponds to a special case of his general model (1), (2) in d’Onofrio (2008)), with ϕ(x)=k, f(x)=a(1−bx), β(x)=0, q(x)=1 and μ(x)=(d+|m|·x). As in the models investigated in this study, d’Onofrio has observed that his generalized model admits a cancer-free state, and also predicted that it may attain multiple stable equilibria, which he interpreted as microscopic steady states (MISS) and which we interpret in the context of cancer dormancy. Our own results thus confirm some of d’Onofrios, but go further to explore how specific cancer-immune system interaction models are concretely affected by changing dynamical properties that may be tailored for immunotherapy. In particular, we wanted to explore some of the properties of CISI models that underpin the mechanisms that may give rise to bistability patterns (the aforementioned dominance of immunosuppressive effects). In our models, we were therefore more interested in explicit analytical results, which would allow us to study how bistability patterns depend on effector cell killing efficacy. We also extended this approach to include how adoptive transfer might function under conditions with stochasticity.

A similarly comprehensive analysis of how CISI models may give rise to successful adoptive immunotherapy treatments has recently been put forth by Talkington et al. (2018). Similarly to our own conclusions, Talkington et al. identify bistability as a major prerequisite for successful adoptive immunotherapy. Their approach is also to review a series of models of increasing complexity, whereby complexity is understood to represent incorporations of additional aspects of the immune system, such as helper cells, interleukin and naïve T cells into a base model. The base model is Kuznetsov et al.’s early model from 1994 (Kuznetsov et al., 1994), which, akin to our base model, assumes only two compartments: tumor and effector cells. The Kuznetsov model allows for bistability, with a stable state of the tumor close to cancer eradication. For all other models, Talkington et al. show that when they can give rise to bistability, adoptive immunotherapy leads to successful outcomes in simulations.

Future work could address whether the mechanism for bistability emergence identified in this study, the dominance of immunosuppression by cancer over immune cell proliferation, is also the one that gives rise to bistability in the models examined by Talkington et al. To this end, it will be useful to embark on a more comprehensive analysis of how mathematical models as the ones put forth here, bring about some behavior of interest, such as bistability. One approach that could be taken in this direction follows the axiomatic modeling framework pioneered by Komarova et al. (2003) and (d’Onofrio, 2008, d’Onofrio, 2005).

In a departure from the work of de Pillis et al. (2005), we have concentrated on model features typically used in HIV modeling, borrowing in particular from the study of (Conway and Perelson, 2014). The reason for this choice is that ODE-based modeling has a long tradition in HIV and virus dynamics modeling (Wodarz, 2007, Nowak, May, 2000, Perelson, Ribeiro, 2013). A great wealth of data have helped to validate interaction terms of different cell types in mixtures, and particularly, how CTLs kill. In our view, these advantages can be fruitfully employed in cancer modeling as well. A more inter-disciplinary integration, in particular with respect to CTL behavior, will benefit both fields, and allow for the analysis of structural similarities between models that might be harnessed for immunotherapy design.

Our models show that biologically plausible cancer-immune system interactions may be utilized to induce cancer-free states. Increases in the killing efficacy of immune effector cells can destroy the bistability pattern inherent in those models, abruptly removing the basis for cancer growth.

## 5 Funding

This work was supported by the European Research Council Advanced Grant [grant number PBDR 268540]; the Swiss National Science Foundation [grant number P2EZP3_162257]; and SystemsX the Swiss Initiative for Systems Biology [grant number 51FSP0_163566]. F.F would like to acknowledge the NIH COBRE Program(grant no. 1P20GM130454) and the Bill & Melinda Gates Foundation (award no. OPP1217336) for generous financial support.

## CRediT authorship contribution statement

**Victor Garcia:** Conceptualization, Investigation, Methodology, Formal analysis, Writing - original draft, Writing - review & editing. **Sebastian Bonhoeffer:** Conceptualization, Funding acquisition, Project administration, Supervision, Resources, Writing - original draft, Writing - review & editing. **Feng Fu:** Conceptualization, Investigation, Methodology, Formal analysis, Funding acquisition, Project administration, Supervision, Resources, Writing - original draft, Writing - review & editing.

## Figures and Tables

**Table 2:** Parameter values for the extended base model with saturation (10), (11).

Parameter	Description	Value
*a*	maximum replication rate of unmutated tumor cells (de Pillis et al., 2005)	0.514 ${\mathrm{day}}^{-1}$
*b*	inverse carrying capacity of tumor (de Pillis et al., 2005)	$1.02\cdot 10^{-9}$${\mathrm{cells}}^{-1}$
*k*	killing efficiency of effector cells or CTLs (Ganusov, Goonetilleke, Liu, Ferrari, Shaw, McMichael, Borrow, Korber, Perelson, 2011, Wick, Yang, Corey, Self, 2005, Elemans, Florins, Willems, Asquith, 2014, de Pillis, Radunskaya, Wiseman, 2005)	$10^{-5}-10^{-3}$${\mathrm{cells}}^{-1}{\mathrm{day}}^{-1}$
*σ*	replenishment rate of effector cells or CTLs *E* (Althaus, De Boer, 2008, De Boer, 2007)	5 · 10^3^$\mathrm{cells}\cdot {\mathrm{day}}^{-1}$
*d*	effector or CTL loss rate (Conway and Perelson, 2014)	2 ${\mathrm{day}}^{-1}$
*d_e_*	effector CTL immune impairment rate	$10^{-3}-10^{3}$${\mathrm{day}}^{-1}$
*b_e_*	maximum CTL proliferation rate (Davenport, Ribeiro, Chao, Perelson, 2004, Conway, Perelson, 2014)	1 ${\mathrm{day}}^{-1}$
*κ_e_*	saturation coefficient of effector immune growth	5 · 10^2^ cells
*κ_d_*	saturation coefficient of effector immune exhaustion	2.5 · 10^4^ cells

**Table 3:** Parameter values for the extended model with saturation in NK and CTL (21), (22), (23).

Parameter	Description	Value
*a*	maximum replication rate of unmutated tumor cells (de Pillis et al., 2005)	0.514 ${\mathrm{day}}^{-1}$
*b*	inverse carrying capacity of tumor (de Pillis et al., 2005)	$1.02\cdot 10^{-9}$${\mathrm{cells}}^{-1}$
*c*	killing efficacy natural killer cells	$10^{-7}-10^{0}$${\mathrm{cells}}^{-1}{\mathrm{day}}^{-1}$
*k*	killing efficiency of effector cells or CTLs (Ganusov, Goonetilleke, Liu, Ferrari, Shaw, McMichael, Borrow, Korber, Perelson, 2011, Wick, Yang, Corey, Self, 2005, Elemans, Florins, Willems, Asquith, 2014)	$10^{-5}-10$${\mathrm{cells}}^{-1}{\mathrm{day}}^{-1}$
*σ*	replenishment rate of effector cells or CTLs *E* (Althaus, De Boer, 2008, De Boer, 2007)	$10-10^{3}$$\mathrm{cells}\cdot {\mathrm{day}}^{-1}$
*b_n_*	proliferation rate of natural killer cells *N*	1.5 ${\mathrm{cells}}^{-1}{\mathrm{day}}^{-1}$
*d_n_*	exhaustion rate of natural killer cells *N*	1 ${\mathrm{cells}}^{-1}{\mathrm{day}}^{-1}$
*μ*	death rate of natural killer cells *N*	1 ${\mathrm{day}}^{-1}$
*d*	effector loss rate (Conway and Perelson, 2014)	2 ${\mathrm{day}}^{-1}$
*d_e_*	CTL immune impairment rate (Johnson, Kochin, McAfee, Stromnes, Regoes, Ahmed, Blattman, Antia, 2011, Conway, Perelson, 2014)	2 ${\mathrm{day}}^{-1}$
*b_e_*	maximum CTL proliferation rate (Davenport, Ribeiro, Chao, Perelson, 2004, Conway, Perelson, 2014)	1 ${\mathrm{day}}^{-1}$
*ω*	growth stimulation rate of cytotoxic T lymphocytes *E* by natural killer cells (de Pillis et al., 2005)	$1.1\cdot 10^{-7}$${\mathrm{cells}}^{-1}{\mathrm{day}}^{-1}$
*κ_be_*	saturation coefficient of CTL immune growth	5 · 10^2^ cells
*κ_de_*	saturation coefficient of CTL immune exhaustion	2.5 · 10^4^ cells
*κ_bn_*	saturation coefficient of NK immune growth	10^4^ cells
*κ_dn_*	saturation coefficient of NK immune exhaustion	10^3^ cells

## Appendix A Mathematical analysis of the base model

Here, we describe the analysis of the system of (1), (2) and its equilibria (termed *E** and *T** in the following). To this end, we first solve dE/dt=0 for *E**, and obtain an expression in *T**. We then subsitute *E** in $\frac{dT^{*}}{dt},$ and obtain a polynomial in *T**. We aim to find the roots of $\frac{dT^{*}}{dt}\left(T^{*}\right),$ which are the fixed points of the system of equations. Removing the one obvious fixed point given by $\left(T_{1}^{*},E_{1}^{*}\right)=\left(0,\sigma /d\right),$ we are left with a quadratic equation in *T**. To obtain the other fixed points, we analyze its roots:

(25) $$\frac{dT^{*}}{dt}=F\left(T^{*},E^{*}\right)=abmT^{*2}-a\left(m+bd\right)T^{*}+\left(ad-k\sigma \right)=0$$

The solutions of (25) are given in (3).

With this, we can derive the exact conditions for (i) both $T_{2}^{*}$ and $T_{3}^{*}$ below zero (ii) $T_{2}^{*}$ and $T_{3}^{*}$ of different signs and (iii) both $T_{2}^{*}$ and $T_{3}^{*}$ above zero.

We go through all cases, starting with case i). For both solutions $T_{2,3}^{*}$ to be negative, we have two subcases ia) and ib) of sets of conditions. Either (ia), we require that a(m+bd)<0, $\sqrt{\Delta_{s}}<\left|a\left(m+bd\right)\right|,$ $\sqrt{\Delta_{s}}>0$ and *abm* > 0, or equivalently (ib), a(m+bd)>0, $\sqrt{\Delta_{s}}<\left|a\left(m+bd\right)\right|,$ $\sqrt{\Delta_{s}}>0$ and *abm* < 0.

We want to investigate the conditions that are equal in both sub-cases to simplify the expressions that lead to i). Let us start by analyzing the condition $\sqrt{\Delta_{s}}<\left|a\left(m+bd\right)\right|,$ which is the same in both sub-cases. This is equivalent to stating that 4abm(ad−kσ)>0. In this expression, *a, b, d* and *σ* have to be assumed to be positive for biological reasons. *k*, the net effector cell killing of cancer cells can theoretically become negative, which could be the consequence of cancer growth stimulation by the presence of effector cells as reported in some recent studies (Mellman et al., 2011). The net growth stimulation from cancer cells *m* could theoretically become negative also, since cancer cells are known to evolve mechanisms that activate suppressive T regulatory cells and thus escape effector cell action (Mellman et al., 2011). This immunosuppressive effect could overpower the growth stimulation of T cells induced by the presence of cancer. Thus, for 4abm(ad−kσ)>0 to be valid, there exist two options: Either ad−kσ>0 and *m* > 0, or ad−kσ<0 and *m* < 0.

The other condition that appears in both sub-cases is Δ_*s*_ > 0, which is required to obtain real solutions for $T_{2,3}^{*}$. Depending on the sign of *m*, this translates into different conditions for *k*: if *m* > 0, then $k>\left(ad-{\left(a\left(m+bd\right)\right)}^{2}/4abm\right)/\sigma ,$ and otherwise if *m* < 0, then $k<\left(ad-{\left(a\left(m+bd\right)\right)}^{2}/4abm\right)/\sigma$.

These conditions on *m* must concord with the conditions on *abm* derived before, since *a, b* > 0. And thus if *m* > 0, we are in the same sub-case as a(m+bd)<0 and *abm* > 0, whereas *m* < 0 must coincide with a(m+bd)>0 and *abm* < 0.

Thus, to obtain $T_{2,3}^{*}<0,$ we have two sets of conditions dependent on parameter *m*. The first set of conditions is:

(26) m>0

(27) m+bd<0

(28) ad−kσ>0

(29) $$k>\left(ad-\frac{{\left(a\left(m+bd\right)\right)}^{2}}{4abm}\right)\frac{1}{\sigma },$$

whereas the second set of conditions reads:

(30) m<0

(31) m+bd>0

(32) ad−kσ<0

(33) $$k<\left(ad-\frac{{\left(a\left(m+bd\right)\right)}^{2}}{4abm}\right)\frac{1}{\sigma }.$$

Case ii) is of greater interest, since it entails that there will be one other positive solution to (1), (2). As in case i), there exist two situations in which $T_{2}^{*}<0$ and $T_{3}^{*}>0$ is attained. For these conditions to be both true, it follows that either a(m+bd)>0, $\sqrt{\Delta_{s}}>\left|a\left(m+bd\right)\right|,$ $\sqrt{\Delta_{s}}>0$ and *abm* > 0, or on the other hand, a(m+bd)<0, $\sqrt{\Delta_{s}}>\left|a\left(m+bd\right)\right|,$ $\sqrt{\Delta_{s}}>0$ and *abm* < 0. Note that compared to i), the inequality that compares |a(m+bd)| with $\sqrt{\Delta_{s}}$ has been inverted. Fortunately, again, the inequalities on |a(m+bd)| are equivalent in both sub-cases, so we can proceed analyzing it.

Following an analogous discussion as in i), it follows that for $\sqrt{\Delta_{s}}>\left|a\left(m+bd\right)\right|$ to be true requires either *m* < 0 and ad−kσ>0, or conversely, *m* > 0 and ad−kσ<0. The conditions on *m* must again match the previous conditions on *abm*, since *a, b* > 0. With an analogous reasoning for $\sqrt{\Delta_{s}}>0,$ we ultimately obtain:

(34) m>0

(35) m+bd>0

(36) ad−kσ<0⟺k>ad/σ

(37) $$k>\left(ad-\frac{{\left(a\left(m+bd\right)\right)}^{2}}{4abm}\right)\frac{1}{\sigma },$$

or alternatively:

(38) m<0

(39) m+bd<0

(40) ad−kσ>0⟺k<ad/σ

(41) $$k<\left(ad-\frac{{\left(a\left(m+bd\right)\right)}^{2}}{4abm}\right)\frac{1}{\sigma }.$$

The case iii) is of particular interest, since it entails that there could exist two non-negative cancer attractors (stable fixed points of (1), (2)), which are separated by one unstable state. For both solutions $T_{2,3}^{*}$ to be positive, we require that a(m+bd)>0, $\sqrt{\Delta_{s}}<\left|a\left(m+bd\right)\right|,$ $\sqrt{\Delta_{s}}>0$ and *abm* > 0, or equivalently, a(m+bd)<0, $\sqrt{\Delta_{s}}<\left|a\left(m+bd\right)\right|,$ $\sqrt{\Delta_{s}}>0$ and *abm* < 0. Thus, this is identical to case i), except for the fact that the conditions on a(m+bd) are exactly inverted. We can therefore simply adopt the conclusions from the discussions of $\sqrt{\Delta_{s}}<\left|a\left(m+bd\right)\right|$ and $\sqrt{\Delta_{s}}>0$.

As in i), the *m* > 0 sub-case (one of the two possible sub-cases in the discussion of Δ_*s*_ > 0) to obtain $T_{2,3}^{*}>0,$ needs to satisfy the additional conditions a(m+bd)>0 and *abm* > 0. Following the assumptions about the values of *a, b* and *d*, this immediately entails that m+bd>0 and that *m* > 0. The other sub-case (*m* < 0) requires a(m+bd)<0 and *abm* < 0, which by analogy entails that m+bd<0.

Hence, summarizing, for all conditions for $T_{2,3}^{*}>0$ to be satisfied at the same time, there exist two ways in which this can be achieved that crucially depend on the sign of the effector cell growth stimulation parameter *m*. On the one hand, we will obtain two positive solutions to (1), if:

(42) m>0

(43) m+bd>0

(44) ad−kσ>0

(45) $$k>\left(ad-\frac{{\left(a\left(m+bd\right)\right)}^{2}}{4abm}\right)\frac{1}{\sigma },$$

or otherwise, if:

(46) m<0

(47) m+bd<0

(48) ad−kσ<0

(49) $$k<\left(ad-\frac{{\left(a\left(m+bd\right)\right)}^{2}}{4abm}\right)\frac{1}{\sigma }.$$

This last set of conditions with *m* < 0 is of particular interest. On the one hand, *m* > 0 leads to biologically dissatisfactory scenarios: The root at $T_{1}^{*}=0$ is repulsive, an there exists only one positive attractive root $T_{2}^{*}>0$. This behavior signifies a departure from the concept of an *immunological barrier* (Eftimie, Bramson, Earn, 2010, Kuznetsov, Makalkin, Taylor, Perelson, 1994), whereby a tumor has to first surpass a threshold size, from which it is hindered by the immune system, before being able to grow to large numbers. On the other hand, the resulting bistability pattern in the *m* < 0 case is reminiscent of key features of cancer establishment and growth, which makes them worth studying.

### Stability analysis

To analyze the stability of the equilibria of the system of (1), (2), we perform a classical analysis based on the behavior of the Jacobian of the map *f*: (*E, T*) → (*dE*/*dt, dT*/*dt*) at equilibrium points. To do this, we first interpret (1), (2) as a map:

(50) $$\frac{dT}{dt}=f_{1}\left(T,E\right)=aT\left(1-bT\right)-kTE,$$

(51) $$\frac{dE}{dt}=f_{2}\left(T,E\right)=\sigma -dE+mET.$$

The Jacobian, *J*, of the map *f* is thus:

(52) $$J=\left(\begin{array}{cc} a(1-bT))-abT-kE & -kT\\ mE & -d+mT \end{array}\right).$$

For an equilibrium point to be stable, the trace of *J, tr*(*J*) needs to be negative, while the determinant of *J, det*(*J*), needs to positive. The trace and determinants of *J* must therefore satisfy:

(53) tr(J)=d+(ab−m)T>0⟺ab>m,

where *T* > 0, *d* > 0,

(54) det(J)=m+bd>2mbT.

The second condition (54) is equivalent to $\frac{m+bd}{2mb}>T$ if *m* > 0, and to $\frac{m+bd}{2mb}<T$ if *m* < 0. If *m* > 0, the largest tumor fixed point is $T_{3}^{*}=\frac{a\left(m+bd\right)+\sqrt{\Delta_{s}}}{2abm},$ from (3). Inserting $T_{3}^{*}$ into (54) shows that the condition cannot be satisfied. However, $T_{3}^{*}$ does satisfy *det*(*J*) < 0 if *m* < 0. Thus, only *m* < 0 scenarios can lead to the largest of the fixed points being stable.

## Appendix B Mathematical analysis of the frequency dependent immune response model

To investigate the steady state solutions for the (10), (11), we first solve for the steady state of *E*, which can be found analytically:

(55) $$E^{*}=\frac{\sigma }{d-b_{e}\frac{T}{\kappa_{e}+T}+d_{e}\frac{T}{\kappa_{d}+T}}.$$

Substituting *E* in the expression for *dT*/*dt* with Eq. (55) reduces the finding of the steady states to a one-dimensional problem in *T**:

(56) $$\frac{dT^{*}}{dt}=aT\left(1-bT\right)-kT\frac{\sigma }{d-b_{e}\frac{T}{\kappa_{e}+T}+d_{e}\frac{T}{\kappa_{d}+T}}=0.$$

$T^{*}=0$ is a trivial root of $\frac{dT^{*}}{dt}=0$. After removing the trivial root from (56), the remaining fixed points of the system (10), (11) correspond to the roots of:

(57) $$a\left(1-bT\right)-k\frac{\sigma }{d-b_{e}\frac{T}{\kappa_{e}+T}+d_{e}\frac{T}{\kappa_{d}+T}}=0.$$

This expression can be converted into a polynomial of third order in *T**. Solving Eq. (57) is thus equivalent to the problem of finding the roots of a cubic equation that is obtained from (57) by eliminating the denominators:

(58) $$\begin{array}{ccc} & & T^{3}ab\left(b_{e}-d_{e}-d\right)\\ & & \;+T^{2}\left(a\left(d-b_{e}+d_{e}\right)-ab\left(d\left(\kappa_{e}+\kappa_{d}\right)-b_{e}\kappa_{d}+d_{e}\kappa_{e}\right))-\sigma k\right)\\ & & \;+T\left(a\left(d\left(\kappa_{e}+\kappa_{d}\right)-b_{e}\kappa_{d}+d_{e}\kappa_{e}\right)-abd\kappa_{e}\kappa_{d}-\sigma k\left(\kappa_{e}+\kappa_{d}\right)\right)\\ & & \;+\kappa_{d}\kappa_{e}\left(ad-\sigma k\right)=0 \end{array}$$

In other words, (58) is equivalent to a cubic equation of the form:

(59) $$f\left(x\right)=Ax^{3}+Bx^{2}+Cx+D=0,$$

where T=x and

(60) $$A=ab\left(b_{e}-d_{e}-d\right),$$

(61) $$B=a\left(d-b_{e}+d_{e}\right)\;-\mathrm{ab}\left(d\left(\kappa_{e}+\kappa_{d}\right)-b_{e}\kappa_{d}+d_{e}\kappa_{e}\right))-\sigma k,$$

(62) $$C=a\left(d\left(\kappa_{e}+\kappa_{d}\right)-b_{e}\kappa_{d}+d_{e}\kappa_{e}\right)-\mathrm{abd}\kappa_{e}\kappa_{d}-\sigma k\left(\kappa_{e}+\kappa_{d}\right),$$

(63) $$D=\kappa_{d}\kappa_{e}\left(\mathrm{ad}-\sigma k\right).$$

The cubic is analytically solvable, but tedious to write out in its standard representation. We thus employ a non-standard representation of these roots to better analyze the systems’ behavior and its biological meaning. The properties of the roots of (60) are largely dependent on the discriminant Δ of the cubic equation, which is defined by:

(64) $$\Delta =18ABCD-4B^{3}D+B^{2}C^{2}-4AC^{3}-27A^{2}D^{2}.$$

If Δ > 0 all three roots are real and distinct, if Δ=0 one root is a multiple root, and if Δ < 0 only one root is real, and the other two are complex.

### B1 Two positive roots of the cubic

With this information, we can make some assertions about the conditions that (59) and Δ must satisfy for bistability to exist. Under bistability, two of the three roots of (58) must be positive. Then, together with the root T=0, there will be three possible roots to produce a bistability effect. The case in which all three are positive will be dicussed next.

Two positive roots of (58) can be attained in two ways: a) Δ=0, with both the double and the single root being positive and b) Δ > 0 and only the smallest root being negative, with the other two roots both being positive.

The case a) is likely biologically irrelevant, since all the coefficients in (59) would have to take values to exactly satisfy Δ=0. In case b) where Δ > 0 and all roots exist, we want to find conditions under which exactly two of the three of the roots are positive. To this end, we draw on the theory of cubic functions and on some of its fundamental results about the nature of roots of cubic functions.

An elegant way to represent the roots of the cubic function is by a trigonometric approach. This approach consists in modifying the standard cubic equation by means of the *Tschirnhaus transformation*, where x=z−B/3A, and *z* is the new variable. The transformation uses the insight that the sum of the roots *x_N_* of any n-order polynomial $a_{n}x^{n}+a_{n-1}x^{n-1}\cdots +a_{1}x+a_{0}$ is given by $-a_{n-1}/na_{n}$. Thus, the Tschirnhaus transformation corresponds to a shift along the x-axis that relocates the cubic such that the sum of the roots lies at the origin (Nickalls, 2006). Expressing the standard cubic representation (59) in terms of the variable *z* leads to the *depressed cubic*:

(65) $$f\left(z\right)=z^{3}+pz+q,$$

where

(66) $$p=\frac{3AC-B^{2}}{3A^{2}}$$

(67) $$q=\frac{2B^{3}-9ABC+27A^{2}D}{27A^{3}}.$$

If three real solutions exist (Δ > 0), the roots of the depressed equation can be expressed by the use of trigonometric functions (Zucker, 2008, Nickalls, 2006). Then, the real roots of the depressed cubic equation are (Beyer and Shelby, 1975):

(68) $$z_{k}=2\sqrt{-\frac{p}{3}}\cos\left(\frac{1}{3}\arccos\left(\frac{3q}{2p}\sqrt{\frac{-3}{p}}\right)-\frac{2\pi k}{3}\right),k=0,1,2.$$

This equation is only valid if the argument of the arccosine of $\theta =\frac{3q}{2p}\sqrt{\frac{-3}{p}}$ is ∈(−1,1). Interestingly, this is equivalent to demanding that $4p^{3}+27q^{2}\leq 0$. This condition implies that *p* < 0, which is also a necessary condition for $\sqrt{-\frac{p}{3}}\in R$. The solution (68) has an elegant geometric interpretation. The roots can be represented as the projections of the vertices of an equilateral triangle with a circle of radius $2\sqrt{-\frac{p}{3}}$ onto the x-axis. The center of the circle is located at *x_N_*.

Thus, what is needed to ensure that one real root is negative and two real roots are positive is to demand that the back-transformation of the smallest root, $\min_{k}\;z_{k}-B/3A$, be smaller than zero, and the next largest back-transformed root be positive. Cubic function theory has produced useful results on how to find the smallest solution $\min_{k}\;z_{k}$ (Nickalls, 2006, Beyer, Shelby, 1975) as well as the position of the other roots in relation to it. Let *z*_0_ be interpreted as a function of *p* and *q*, that is, $z_{0}=C\left(p,q\right)$. Then the three roots can be expressed in terms of *C*(*p, q*), namely

(69) $$z_{0}=C\left(p,q\right),\;z_{2}=-C\left(p,-q\right),\;z_{1}=-z_{0}-z_{2}.\;$$

Furthermore, if the three roots are real, we have *z*_0_ ≥ *z*_1_ ≥ *z*_2_ (Nickalls, 2006, Beyer, Shelby, 1975). Thus, *z*_2_ is the smallest of the solutions, and therefore:

(70) $$\min_{k}\;z_{k}=-2\sqrt{\frac{-p}{3}}\cos\left(\frac{1}{3}\arccos\left(\frac{-3q}{2p}\sqrt{\frac{-3}{p}}\right)\right).$$

With this, the conditions to obtain one negative and two positive roots can be specified. First, Δ > 0 for all roots to be real-valued, second $z_{2}-B/3A<0$ to obtain a negative minimal root, and third, $z_{1}-B/3A>0$ to ensure that the second largest root is positive. When replacing the values of *p* and *q* in (65) with terms of the coefficients of the standard cubic function (59), these conditions read as follows:

(71) $$18ABCD-4B^{3}D+B^{2}C^{2}-4AC^{3}-27A^{2}D^{2}>0$$

(72) $$\begin{array}{ccc} & & -2\sqrt{\frac{B^{2}-3AC}{9A^{2}}}\cos(\frac{1}{3}\arccos(\frac{B\left(2B^{2}-9AC\right)+27A^{2}D}{6A(B^{2}-3AC)}\\ & & \;\sqrt{\frac{9A^{2}}{B^{2}-3AC}}))-\frac{B}{3A}<0 \end{array}$$

(73) $$\begin{array}{ccc} & & 2\sqrt{\frac{B^{2}-3AC}{9A^{2}}}\cos(\frac{1}{3}\arccos\left(\frac{B\left(2B^{2}-9AC\right)+27A^{2}D}{6A(3AC-B^{2})}\sqrt{\frac{9A^{2}}{B^{2}-3AC}}\right)\\ & & \;-\frac{2\pi }{3})-\frac{B}{3A}>0. \end{array}$$

Expressing *A, B, C* and *D* in terms of the parameters of model (10), (11) gives the full solutions to the conditions for bistability. These conditions only ensure a certain position of the roots, but do not establish whether these roots are ordered into stable and unstable equilibria in a biologically reasonable way.

### B2 Three positive roots of the cubic

The trigonometric interpretation of the cubic also allows us to identify the conditions for all roots to be positive. Applying the same reasoning as in the two-positive-roots case (Appendix B.1), we impose that the sum of the smallest root *z_k_* and the shift implied in the Tschirnhaus transformation, $x_{N}=-B/3A,$ is larger than zero. In other words, we ask that the solution *z_k_*, which is located the farthest from *x_N_* in negative-x direction should be smaller than *x_N_* itself. In this case, all roots will come to lie on the positive part of the x-axis, although the circle of radius $2\sqrt{-\frac{p}{3}}$ around *x_N_* might reach into negative x-values.

The condition $\min_{k}\;z_{k}-B/3A>0$ is thus the precise condition for which all roots are positive. By virtue of (70), and inserting the values of *p* and *q* in terms of the coefficients of the standard cubic function (59), the conditions for only positive roots become:

(74) $$18ABCD-4B^{3}D+B^{2}C^{2}-4AC^{3}-27A^{2}D^{2}>0$$

(75) $$\begin{array}{ccc} & & -2\sqrt{\frac{B^{2}-3AC}{9A^{2}}}\cos(\frac{1}{3}\arccos(\frac{B\left(2B^{2}-9AC\right)+27A^{2}D}{6A(B^{2}-3AC)}\\ & & \;\sqrt{\frac{9A^{2}}{B^{2}-3AC}}))-\frac{B}{3A}>0. \end{array}$$

These are implicit inequalities in A, B, C and D, which in turn depend on the parameters of model (10), (11) by means of the relation (60).

Expressing the coefficients A, B, C and D in terms of the parameters of model (10), (11) thus gives the exact analytical conditions under which four non-negative fixed points to (10), (11) exist, out of which three are strictly positive. Writing this expression down is cumbersome and hard to analyze further. However, numerical methods can be employed to examine which regions in parameter space can produce this distinctive pattern of multistability.

### B3 Analyzing parameter space for existence of multistability

We analyzed whether the conditions (71) and (74) are satisfied for realistic parametrizations of model (10), (11). To this end, we fixed most parameters at biologically plausible values found in the literature (see Table 2). We then varied two of those parameters which are either expected to have a large impact on the system’s dynamics, or may be less well understood.

The standard parameter values used for the extended model with saturation are specified in Table 2.

Fig. A1 shows the regions of parameter space whose parameter values lead to bistability as well as multistability in the model (10), (11). The region that supports multistability forms a band across a wide range of immune cell exhaustion rate values *d_e_*. This indicates that when a waning of killing efficacies *k* occurs, the system may trespass this region. A boundary is shared by a region with bistability and the parameter region that only admits a cancer-free state. The mechanism for combination of immunotherapy may thus still be applicable along this boundary, which is present at plausible values of *d_e_* around unity. The rest of the parameter values used for the generation of this plot are within the range of possible values frequently used in cancer modeling.

## Appendix C Mathematical analysis of NK-CTL model

We follow the approach taken for both the base model and the extended base model with saturation and attempt to derive a function $\frac{dT}{dt}\left(T\right)$ to analyze the roots of (21), (22), (23), which are the fixed points of the system. The steady states of *N* and *E* can be found analytically:

(76) $$N^{*}=\frac{\sigma }{\mu -b_{n}\frac{T}{\kappa_{bn}+T}+d_{n}\frac{T}{\kappa_{dn}+T}},$$

whereas

(77) $$E^{*}=\frac{\omega NT}{d-b_{e}\frac{T}{\kappa_{be}+T}+d_{e}\frac{T}{\kappa_{de}+T}}.$$

Substituting *N* and *E* in the expression for $\frac{dT}{dt}\left(T\right)$ in (21), (22), (23) reveals that $\frac{dT}{dt}\left(T\right)$ can be expressed as

(78) $$\frac{dT}{dt}\left(T,\frac{dE}{dt}=0,\frac{dN}{dt}=0\right)=\frac{P(T)}{Q(T)}.$$

Here, P(T)=F(T)T is a sixth-order polynomial in *T*. Again removing the trivial solution $T_{1}^{*}=0,$ leaves a polynomial *F*(*T*) of fifth order. *Q*(*T*) is a fourth order polynomial. The roots of *P*(*T*) constitute the remaining fixed points of the system. In general, an analytical expression for the roots for such a polynomial can only be given up to the fourth order. Thus, we first restrict ourselves to discussing more general properties that *P*(*T*) and *Q*(*T*) need to satisfy in order to generate biologically plausible situations.

For this, it is important to keep in mind that $\frac{dT}{dt}\left(T,\frac{dE}{dt}=0,\frac{dN}{dt}=0\right)$ has a narrow mathematical interpretation. In the three-dimensional state space spanned by *T, E* and *N*, it describes the behavior of $\frac{dT}{dt}$ along the intersection between the nullclines of *E* and *N*. In a three-dimensional space, this intersection is typically a line passing through a steady state. Imagine a point with some *T*-value approaching a steady state at *T** on the intersecting line. For the steady state to be stable, $\frac{dT}{dt}$ would have to be positive as the point, and with it *T* < *T**, reaches *T** from below. Equally, as the point moves past the steady state into larger values *T* > *T**, $\frac{dT}{dt}$ would have to grow negative. We call this a *stabilizing* behavior of $\frac{dT}{dt}$ at the steady state. If a steady state satisfies this condition, we call it *T*-stabilizing. Clearly, this behavior of $\frac{dT}{dt}$ around the steady state does not guarantee that it is stable: instability could still be caused by other properties of the derivatives field perpendicular to the null cline analyzed. However, it is a pre-requisite for the steady state to be stable.

We begin our discussion by analyzing the simpler problem $\frac{dT}{dt}\left(T\right)=F\left(T\right)T$. Here, $\frac{dT}{dt}\left(T\right)$ is a polynomial *F*(*T*) times *T*. The order of the polynomial *F*(*T*) and the sign of the leading coefficient α of that polynomial, influences the locations of the roots of *F*(*T*), and thus also of $\frac{dT}{dt}\left(T\right)$. *F*(*T*) also influences how the roots are arranged into stable fixed points.

The *n*th order polynomial will maximally have *n* real valued roots. If *n* is odd, and α < 0, the smallest and largest fixed points of *F*(*T*) are *T*-stabilizing. Conversely, if *n* is odd, and α > 0, the smallest and largest fixed points of *F*(*T*) are unstable. If *n* is even, and α < 0, the smallest fixed point of *F*(*T*) is unstable, while the largest is *T*-stabilizing. Analogously, if *n* is even, and α > 0, the smallest fixed point of *F*(*T*) is *T*-stabilizing, and the largest is unstable.

These claims follow from the intermediate value theorem. If *n* is odd, the limits of the polynomial for *T* → ∞ and T→−∞ will be of opposite signs. If the leading coefficient α of the polynomial is positive, $\lim_{T\to -\infty }F\left(T\right)=-\infty ,$ and $\lim_{T\to \infty }F\left(T\right)=\infty$. To attain values from −∞ to ∞, *F*(*T*) must traverse the *T*-axis from negative to positive by virtue of the intermediate value theorem. Thus, the first root must be an unstable fixed point. Since there exist an odd number of roots, and the roots must alternate from *T*-stabilizing to unstable, the last root must also be an unstable equilibrium. The converse is true for α < 0, with both, the smallest and largest fixed points being *T*-stabilizing. For even values of *n*, if α > 0, *F*(*T*) will either be ∞ in the limit *T* →  ± ∞ and −∞ for α < 0. Thus, following the same logic as with *n* odd, the smallest fixed point must be *T*-stabilizing if α > 0, while the largest will be unstable. Conversely, if α < 0, the smallest fixed point will unstable, whereas the largest will be *T*-stabilizing.

Multiplying *F*(*T*) with *T* does not alter the position of *F*(*T*)’s roots. However, it will have the effect of adding a new root T=0 to $\frac{dT}{dt}\left(T\right)=F\left(T\right)T,$ and to switch *T*-stabilizing into unstable (and vice versa) fixed points if they are negative.

With this in mind, we can now turn our attention to the full expression, $\frac{dT}{dt}\left(T\right)=\frac{P(T)}{Q(T)}$. Division by *Q*(*T*) does not alter the position of the roots (unless the roots of *Q*(*T*) are identical with the roots of *P*(*T*), in which case they would cancel out). However, it can modify the *T*-stabilizing properties of the roots. Roots will be *T*-stabilizing if the *T*-derivative of $\frac{dT}{dt}\left(T\right)$ is negative. Let us assume that *F*(*T*) has *n* roots *T_i_* (i∈{1,⋯,n}). Then,

(79) $$\frac{d}{dT}\frac{dT}{dt}\left(T\right)=\frac{F\left(T\right)\left(Q\left(T\right)-T\frac{d}{dT}Q\left(T\right)\right)+TQ\left(T\right)\frac{d}{dT}F\left(T\right)}{Q^{2}\left(T\right)}.$$

If we evaluate this expression at a stable root of *F*(*T*), *T_i_*, where $\frac{d}{dT}F\left(T\right)<0,$ we obtain that:

(80) $$\frac{d}{dT}\frac{dT}{dt}\left(T_{i}\right)=\frac{T_{i}Q\left(T_{i}\right)\frac{d}{dT}F\left(T_{i}\right)}{Q^{2}\left(T_{i}\right)}.$$

This entails that the of root *T_i_* > 0 (the only biologically plausible solution) will only be stable if *Q*(*T_i_*) > 0. Thus, for the roots of *F*(*T*) to retain their *T*-stabilizing properties, they will need to come to lie in intervals of *T* where *Q*(*T*) is positive.

Since the fixed points of (21), (22), (23) can be described as *F*(*T*)*T*, the above reasoning is valid, and we can draw conclusions about what conditions the leading coefficient α must satisfy to ensure biologically plausible equilibria. Analyzing the structure of $\frac{dT}{dt}\left(T\right)$ with *Mathematica* (Wolfram Research, 2011), reveals that the coefficient $\alpha =a_{5}$ of the polynomial $F\left(T\right)=a_{5}T^{5}+a_{4}T^{4}+a_{3}T^{3}+a_{2}T^{2}+a_{1}T^{1}+a_{0}$ reads as follows:

(81) $$a_{5}=-ab\left(\left(b_{e}-d_{e}-d\right)\left(b_{n}-\mu -d_{n}\right)\right)-k\sigma \omega .$$

Given that the order of the polynomial *F*(*T*) in NK-CTL model (21), (22), (23) is odd, the leading coefficient *a*_5_ of the polynomial must be negative and the value of *Q*(*T*) at the largest fixed point positive in order for *F*(*T*) to have a *T*-stabilizing largest positive fixed point. Thus, analogously to the situation in the extended base model with saturation, the biological plausibility of the positioning of the fixed points is compatible with *a*_5_ < 0. Again, *a*_5_ expresses the balance between proliferative and suppressive forces acting on NK as well as CTL cells, and is independent from the saturation coefficients. As in the previous models, we assume that *a, b, d, σ, k* > 0 and it is biologically reasonable also to assume *ω* > 0. Thus, interestingly, *a*_5_ < 0 can be satisfied by increasing *kσω*. But if *kσω* is negligible, *a*_5_ will only be negative if $\left(b_{e}-d_{e}-d\right)$ and $\left(b_{n}-\mu -d_{n}\right)$ are both negative or both positive. Thus, the NK-CTL model allows for more flexibility to attain biologically plausible scenarios than the two previous models: if the balance between proliferative and suppressive forces in both NK and CTLs is tipped in favor of exhaustion of the immune cells, biologically interpretable dynamics can emerge. This is analogous behavior to an *m* < 0 situation in the base model. Similarly, if the balance is tipped towards proliferation in both cell types, the arising scenarios are again biologically sound. However, the behavior between both immune cell types must be similar to attain this.

Unfortunately, the above analysis does not reveal whether bi- or multistability can be guaranteed to arise for special combinations of parameters. To show this, we must fall back to numerical methods. In the following, we prove that the system (21) can generate at least bistability patterns of the kind displayed by the base model. We only analyzed the effects of changing *c*, the killing efficacy of NKs rather than CTLs, on the system. We chose *c* because NK have been estimated to have faster cancer suppressing effects than CTLs in de Pillis et al. (2005). Fig. A2 shows the emergence of the bistability pattern for biologically sound parameter choices in the model (21), (22), (23). The parameter values are specified in Table 3.
